# Efficacy, safety and clinical outcome associated with statin use for primary prevention in Korean patients with low-density lipoprotein cholesterol level ≥ 190 mg/dL: A retrospective cohort study

**DOI:** 10.1371/journal.pone.0280586

**Published:** 2023-06-12

**Authors:** Hack-Lyoung Kim, Woo-Hyun Lim, Jae-Bin Seo, Joo-Hee Zo, Myung-A Kim, Sang-Hyun Kim

**Affiliations:** Division of Cardiology, Department of Internal Medicine, Boramae Medical Center, Seoul National University College of Medicine, Seoul, Korea; Department of Public Health of Federal District / Health Sciences Teaching and Research Foundation, BRAZIL

## Abstract

**Background:**

Although the current guideline recommends the use of high-intensity statin to reduce the low-density lipoprotein cholesterol (LDL-C) level by 50% in patients with baseline value of ≥ 190 mg/dL, direct application of this recommendation to Asian populations is still questionable. This study was performed to investigate the statin response of LDL-C in Korean patients with LDL-C ≥ 190 mg/dL.

**Methods:**

A total of 1,075 Korean patients (age 60.7 ± 12.2 years, women 68%) with baseline LDL-C ≥ 190 mg/dL without cardiovascular disease was retrospectively reviewed. Lipid profiles at 6 months, side effects and clinical outcomes during the follow-up period after statin treatment were assessed according to statin intensity.

**Results:**

Most of the patients (76.3%) were treated with moderate-intensity statins, 11.4% with high-intensity statins, and 12.3% with a statin + ezetimibe. The reductions in LDL-C percentage at 6 months were 48.0%, 56.0% and 53.3% in patients treated with moderate-intensity statins, high-intensity statins and statin + ezetimibe, respectively (*P* < 0.001). Side effects requiring dose reduction, medication switch or drug interruption were observed in 1.3%, 4.9% and 2.3% of patients treated with moderate-intensity statin, high-intensity statin and statin + ezetimibe, respectively (*P* = 0.024). During the median follow-duration of 815 days (interquartile range, 408–1,361 days), the incidences of cardiovascular events were not different among the 3 groups (log-rank *P* = 0.823).

**Conclusions:**

Compared to high-intensity statin, moderate-intensity statin was effective enough in reaching target goal of LDL-C without increase in cardiovascular risk and with fewer side effects in Korean patients with LDL-C ≥ 190 mg/dL.

## Introduction

Low-density lipoprotein cholesterol (LDL-C) is a well-established risk factor for cardiovascular disease (CVD), and the clinical benefit of lowering LDL-C with statin is confirmative [1–4]. The current guideline recommends the use of high-intensity statin to reduce LDL-C level by 50% in patients with baseline value of ≥ 190 mg/dL [5]; however, direct application of this recommendation to Asian populations is still questionable. Several Asian studies have shown that less aggressive LDL-C-lowering therapy can be sufficient to produce a substantial and beneficial risk reduction for the prevention of CVD [6–13]. More specifically, it has been reported that LDL-C reduction with rosuvastatin 10 mg in Chinese was significantly greater than in Western people (-52.8% versus -40.9% to -49.7%) [14]. The same study also showed that to achieve LDL-C > 40%, Westerners required atorvastatin 80 mg or rosuvastatin 20 mg, while Asians only needed atorvastatin 19 mg and rosuvastatin 14 mg [14]. However, the range of LDL-C levels of subjects in those studies showing statin effects in Asians was vast. Data indicating whether Asian patients with LDL-C ≥ 190 mg/dL also achieve similar benefits as Western patients at lower statin dose has scarcely been reported [7]. This study was performed to investigate the statin response of LDL-C in Korean patients with baseline LDL-C ≥ 190 mg/dL. Clinical outcome according to statin types was also assessed.

## Methods

### Study population

This single center retrospective cohort study was performed at Boramae Medical Center, a general hospital in Seoul, South Korea. Between January 2010 and March 2016, a total of 4,016 subjects having LDL-C ≥ 190 mg/dL at baseline without cholesterol-lowering medications and without documented cardiovascular disease were identified using a computerized record inquiry system. In order to rule out the possibility of the secondary cause of hypercholesterolemia, 905 subjects with triglyceride ≥ 200 mg/dL were excluded. In addition, 1,396 subjects were also excluded due to thyroid and chronic liver disease, the use of lipid-lowering medications other than statin, including fibrate and omega-3 fatty acid, or follow-up loss at 6 months after statin treatment. Therefore, the remaining 1,075 subjects were included in this study, and their medical records were retrospectively reviewed. All study subjects received statin or statin combined with ezetimibe. The study protocol was reviewed by Institutional Review Board (IRB) of Boramae Medical Center (Seoul, Korea) (IRB number, 26-2016-181). Informed consent was waived by IRB due to retrospective design and routine nature of information collected.

### Data collection

Demographic characteristics along with clinical data were obtained at baseline. Body mass index was calculated as weight (kg)/height (m^2^). Hypertension and diabetes mellitus were defined based on the previous diagnosis and current medications controlling them. Additionally, hypertension was defined as blood pressure > 140/90 mmHg, and diabetes mellitus was defined as fasting plasma glucose level > 126 mg/dL. A subject who smoked regularly within the previous 12 months was considered a current smoker. Ischemic heart disease included acute myocardial infarction and coronary revascularization. Stroke was defined as a neurologic deficit with evidence of infarction or hemorrhage by brain imaging. Laboratory data obtained from venous blood after overnight fasting were also available, such as white blood cell count, hemoglobin, glucose, glycated hemoglobin (HbA1c), total cholesterol (TC), low-density lipoprotein cholesterol (LDL-C), high-density lipoprotein cholesterol (HDL-C), aspartate aminotransferase (AST), alanine aminotransferase (ALT), creatinine and C-reactive protein. Estimated glomerular filtration rate (GFR) was calculated using the Diet in Renal Disease Study (MDRD) equation.

### Cholesterol lowering medications

Seven types of statins currently available in Korea were selected for this study, and those medications were classified into 16 categories according to their dosage: atorvastatin (10, 20, 40 and 80 mg), rosuvastatin (5, 10 and 20 mg), pitavastatin (2 and 4 mg), pravastatin (40 mg), simvastatin (20 and 40 mg), fluvastatin (80 mg), simvastatin + ezetimibe (10 + 10 mg and 20 + 10 mg), and atorvastatin + ezetimibe (10 + 10 mg). Statin intensity was classified into moderate- and high-intensity according to the current guideline, and study subjects were classified into 3 groups according to their statin medications: 1) the moderate-intensity statin group (atorvastatin 10–20 mg, rosuvastatin 5–10 mg, simvastatin 20–40 mg, pravaststatin 40 mg, fluvastatin 80 mg and pitavastatin 2–4 mg), 2) the high-intensity statin group (atorvastatin 40–80 mg and rosuvastastin 20 mg), and 3) the statin + ezetimibe group [simvastatin + ezetimibe (10 + 10 mg and 20 + 10 mg), and atorvastatin + ezetimibe (10 + 10 mg)] [6].

### Assessment of side effects

Side effects of statin medications were reviewed. The side effects caused by statins were defined when they met the following conditions: 1) occurrence during statin use, 2) improvement when statins were reduced or stopped, and 3) no other clear causes to explain the side effects.

### Clinical outcomes

The primary study end-point was a composite of all-cause death, non-fatal myocardial infarction and non-fatal ischemic stroke. Myocardial infarction was defined as elevation in cardiac troponin values with at least 1 value above the 99th percentile upper reference limit, with symptoms of myocardial ischemia, new ischemic electrocardiography changes, development of pathologic Q waves, or imaging evidence of myocardial infarction. Ischemic stroke was defined based on a focal neurologic deficit lasting more than 24 hours with evidence of infarction of brain tissue in imaging study. Clinical follow-up was done every 3 to 6 months and whenever clinical event took place. If clinical follow-up could not be done for more than 6 months, we obtained data on death from the Ministry of the Interior and Safety of Korea. All events were identified by a physician in charge and confirmed by the principal investigator.

### Statistical analysis

All numeric data are expressed as mean ± standard deviation for continuous variables and percentage for discrete variables. Study subjects were divided into 3 groups according to the statin therapy (moderate-intensity statin *vs*. high-intensity statin *vs*. statin plus ezetimibe), and the differences in clinical characteristics and laboratory findings among the 3 groups were compared using analysis of variance (ANOVA) for continuous variable and the chi-square test for discrete variables. Comparisons of variables between pre- and post-treatment of statin were made using the paired *t* tests. Survival and event-free survival rates among the groups were compared using Kaplan-Meier survival analysis with log-rank test. A *P* value of < 0.05 indicated statistical significance. All statistical tests were performed by using SPSS for Windows version 22 (IBM Co., Armonk, NY, USA).

## Results

**Fig 1:**
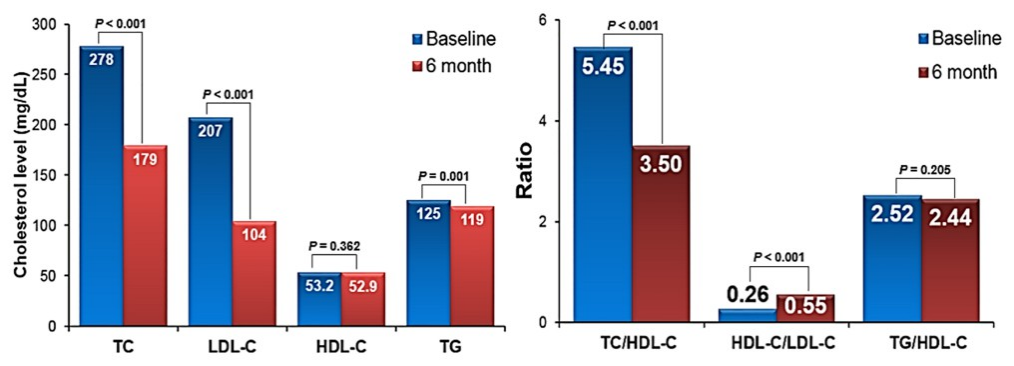
Changes of blood cholesterol profiles after 6-month treatment of statin or statin + ezetimibe. TC, total cholesterol; LDL-C, low-density lipoprotein cholesterol; HDL-C, high-density lipoprotein cholesterol; TG, triglyceride.

**Fig 2:**
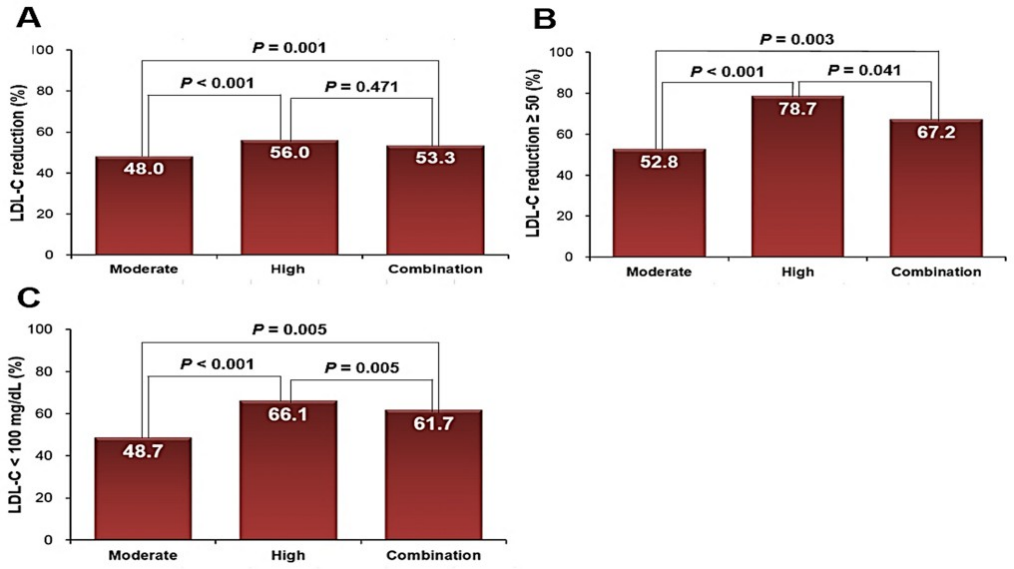
LDL-C response by 6-month treatment of statin or statin + ezetimibe. LDL-C, low-density lipoprotein cholesterol.

**Fig 3:**
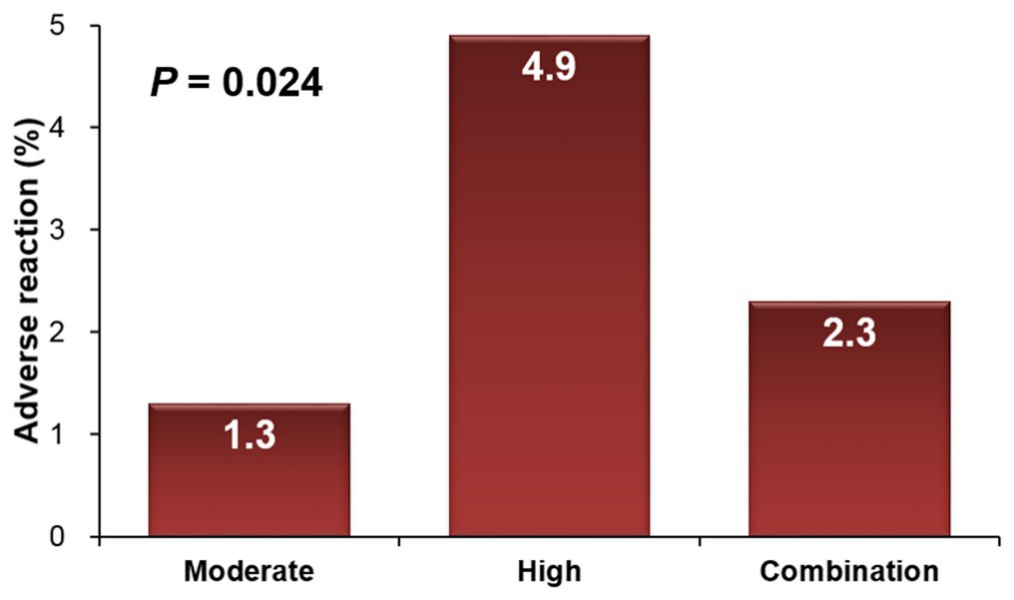
Adverse reaction of statin or statin + ezetimibe.

**Fig 4:**
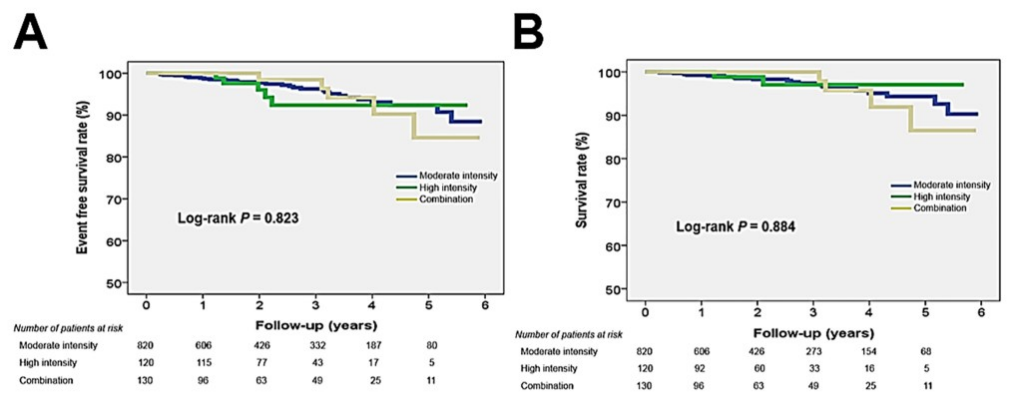
Clinical outcome according to statin intensity.

Names and doses of initial statins used in this study patients are listed in Table 1. In total study patients (n = 1,075), mean age was 60.7 ± 12.2 years, 68% were female and baseline LDL-C was 207 ± 21 mg/dL. The majority of patients (76.3%) were taking moderate-intensity statins, and only 150 (11.4%) and 145 (12.3%) patients were taking high intensity statins + ezetimibe, respectively. Table 2 shows baseline clinical characteristics of the study patients according to statin types. Most of the clinical parameters, such as age, sex, body mass index and cardiovascular risk factors, were not different among the 3 groups except a higher prevalence of hypertension in the statin + ezetimibe group. In cholesterol profiles, TC (275 ± 24 mg/dL in moderate-intensity statin group; 291 ± 37 mg/dL in high-intensity statin group; 284 ± 40 mg/dL in statin + ezetimibe group; P < 0.001) and LDL-C (205 ± 16 mg/dL in moderate-intensity statin group; 217 ± 29 mg/dL in high-intensity statin group; 211 ± 33 mg/dL in statin + ezetimibe group; *P* < 0.001) were higher in the high-intensity and statin + ezetimibe groups compared to moderate-intensity statin group. HDL-C, TG, HDL-C/LDL-C and TG/HDL-C were not different among the 3 groups. Glucose profiles as well as hepatic and renal functions were also similar among the 3 groups. Changes in cholesterol profiles after 6 months of statin treatment are demonstrated in Table 3 and Fig 1. The levels of total cholesterol and LDL-C were significantly decreased after statin treatment in all 3 groups (205 ± 16 mg/dL to 106 ± 31 mg/dL [*P* < 0.001] in moderate-intensity statin group; 217 ± 29 mg/dL to 95 ± 31 mg/dL [*P* < 0.001] in high-intensity statin group; 211 ± 33 mg/dL to 99 ± 34 mg/dL [*P* < 0.001] in statin + ezetimibe group). The reductions in LDL-C percentage at 6 months were 48.0%, 56.0% and 53.3% in patients treated with moderate-intensity statins, high-intensity statins and statin + ezetimibe, respectively (*P* < 0.001 for each). The HDL-C level did not change in either of the 3 groups. The triglyceride level was significantly decreased in the moderate- and high-intensity statin groups, but not in the statin + ezetimibe group. The total cholesterol/HDL-C ratio (5.40 ± 1.24 to 3.56 ± 0.92 [*P* < 0.001] in moderate-intensity statin group; 5.64 ± 1.40 to 3.33 ± 0.91 [*P* < 0.001] in high-intensity statin group; 5.54 ± 1.41 to 3.35 ± 0.81 [*P* < 0.001] in statin + ezetimibe group) was significantly decreased, and HDL-C/LDL-C ratio (0.26 ± 0.10 to 0.54 ± 0.28 [*P* < 0.001] in moderate-intensity statin group; 0.26 ± 0.18 to 0.61 ± 0.21 [*P* < 0.001] in high-intensity statin group; 0.25 ± 0.07 to 0.59 ± 0.22 [*P* < 0.001] in statin + ezetimibe group) was significantly increased in all 3 groups. TG/HDL-C did not change in either of the 3 groups. LDL-C reduction at 6 months are demonstrated in Fig 2. Compared to the moderate-intensity statin group (mean LDL-C reduction: 48.0 ± 15.3 mg/dL), mean LDL-C reductions were significantly greater in the high-intensity statin (56.0 ± 13.1 mg/dL) and statin + ezetimibe group (53.3 ± 15.4 mg/dL) (*P* < 0.05 for each). Proportion of patients with LDL-C reduction ≥ 50% was significantly higher in high intensity statin group (78.7%) than in moderate intensity statin group (52.8%) and ezetimibe combination group (67.2%). Proportion of patients with LDL-C ≥ 100 mg/dL was significantly higher in high intensity statin group (66.1%) than in moderate intensity statin group (48.7%) and ezetimibe combination group (61.7%). Only few patients had side effects associated with statin use (Table 4). The incidence of relatively well-known statin side effects, muscle symptom and liver enzyme elevation, was also very low, and it was not different among the 3 groups (0.4% in moderate-intensity statin group, 0.8% in high-intensity statin group and 1.5% in statin + ezetimibe group; *P* = 0.238). The incidences of all side effects associated with statin use were higher in the high-intensity statin group (*P* = 0.024) (Fig 3). During the median clinical follow-up duration of 815 days (interquartile range, 408–1,361 days), there were 41 cases of composite clinical events (3.8%): 30 cases of death, 2 cases of non-fatal myocardial infarction, and 9 cases of non-fatal ischemic stroke. Event-free survival rate (log-rank *P* = 0.823) and survival rate (log-rank *P* = 0.884) were similar among the 3 groups (Fig 4).

**Table 1:** Statin names and doses.

Statin name	Dose (mg)	n
*Moderate intensity (n = 820*, *76*.*3%)*		
Atorvastatin	10	142
Atorvastatin	20	287
Rosuvastatin	5	30
Rosuvastatin	10	194
Simvastatin	20	33
Simvastatin	40	3
Pravastatin	40	26
Fluvastatin	80	15
Pitavastatin	2	69
Pitavastatin	4	21
*High intensity (n = 123*, *11*.*4%)*		
Atorvastatin	40	32
Atorvastatin	80	5
Rosuvastatin	20	86
*Statin + ezetimibe (n = 132*, *12*.*3%)*		
Simvastatin/ezetimibe	10/10	33
Simvastatin/ezetimibe	20/10	95
Atorvastatin/ezetimibe	10/10	2

**Table 2:** Baseline clinical characteristics of study patients.

Characteristic	Total (n = 1,075)	Moderate intensity (n = 820)	High intensity (n = 123)	Ezetimibe combination (n = 132)	*P* value
Age, years	60.7 ± 12.2	60.5 ± 12.1	61.2 ± 11.7	61.6 ± 12.7	0.537
Female sex, %	732 (68.1)	556 (67.8)	78 (63.4)	98 (74.2)	0.168
Body mass index, kg/m^2^	24.5 ± 3.3	24.6 ± 3.3	24.2 ± 3.4	23.8 ± 3.2	0.134
Comorbidities, %					
Hypertension	383 (35.6)	287 (35.0)	39 (31.7)	57 (43.2)	0.119
Diabetes mellitus	199 (18.5)	149 (18.2)	28 (22.8)	22 (16.7)	0.399
Smoking	106 (9.9)	78 (9.5)	12 (9.8)	16 (12.1)	0.204
Laboratory findings					
Fasting glucose, mg/dL	115 ± 43	117 ± 47	105 ± 28	106 ± 19	0.401
Glycated hemoglobin, %	6.62 ± 1.77	6.62 ± 1.82	6.64 ± 1.71	6.49 ± 1.62	0.887
Total cholesterol, mg/dL	278 ± 29	275 ± 24	291 ± 37	284 ± 40	< 0.001
LDL cholesterol, mg/dL	207 ± 21	205 ± 16	217 ± 29	211 ± 33	< 0.001
HDL cholesterol, mg/dL	53.2 ± 12.0	53.9 ± 11.7	53.6 ± 12.9	53.7 ± 13.5	0.724
Triglyceride, mg/dL	125 ± 38	125 ± 38	125 ± 37	128 ± 39	0.662
Total cholesterol/HDL ratio	5.45 ± 1.28	5.40 ± 1.24	5.64 ± 1.40	5.55 ± 1.41	0.098
HDL/LDL ratio	0.26 ± 0.11	0.26 ± 0.10	0.26 ± 0.18	0.25 ± 0.07	0.817
Triglyceride/HDL ratio	2.52 ± 1.11	2.51 ± 1.11	2.48 ± 1.03	2.57 ± 1.19	0.771
AST, IU/L	24.8 ± 9.5	24.6 ± 9.1	25.7 ± 11.4	25.1 ± 9.9	0.518
ALT, IU/L	24.3 ± 14.0	24.3 ± 14.0	25.5 ± 15.1	23.2 ± 12.4	0.512
eGFR, ml/min/1.73m^2^	85.0 ± 19.1	85.0 ± 19.2	86.1± 19.0	83.7 ± 18.2	0.702
C-reactive protein, mg/L	0.58 ± 2.78	0.59 ± 2.81	0.53 ± 1.75	0.52 ± 3.36	0.941

Numbers are expressed as mean ± standard deviation or n (%). LDL, low-density lipoprotein; HDL, high-density lipoprotein; AST, Aspartate transaminase; ALT, alanine transaminase; eGFR, estimated glomerular filtration rate.

**Table 3:** Changes of cholesterol profiles after 6 months of statin treatment.

Cholesterol profile	Total (n = 1,075)			Moderate intensity (n = 820)			High intensity (n = 123)			Ezetimibe combination (n = 132)		
	Baseline	6 months	*P*	Baseline	6 months	*P*	Baseline	6 months	*P*	Baseline	6 months	*P*
TC, mg/dL	278 ± 29	179 ± 36	< 0.001	275 ± 24	180 ± 35	< 0.001	291 ± 37	171 ± 38	< 0.001	284 ± 40	174 ± 37	< 0.001
LDL-C, mg/dL	207 ± 21	104 ± 32	< 0.001	205 ± 16	106 ± 31	< 0.001	217 ± 29	95 ± 31	< 0.001	211 ± 33	99 ± 34	< 0.001
HDL-C, mg/dL	53.2 ± 12.0	52.9 ± 12.6	0.362	53.9 ± 11.7	52.7 ± 12.1	0.223	53.6 ± 12.9	53.2 ± 13.3	0.893	53.7 ± 13.5	54.1 ± 14.3	0.715
TG, mg/dL	125 ± 38	119 ± 52	0.001	125 ± 38	119 ± 53	0.003	125 ± 37	117 ± 53	0.059	128 ± 39	126 ± 47	0.595
TC/HDL-C	5.45 ± 1.28	3.50 ± 0.91	< 0.001	5.40 ± 1.24	3.56 ± 0.92	< 0.001	5.64 ± 1.40	3.33 ± 0.91	< 0.001	5.56 ± 1.41	3.35 ± 0.81	< 0.001
HDL-C/LDL-C	0.26 ± 0.11	0.55 ± 0.27	< 0.001	0.26 ± 0.10	0.54 ± 0.28	< 0.001	0.26 ± 0.18	0.61 ± 0.21	< 0.001	0.25 ± 0.07	0.59 ± 0.22	< 0.001
TG/HDL-C	2.52 ± 1.11	2.44 ± 1.38	0.057	2.51 ± 1.11	2.44 ± 1.41	0.164	2.48 ± 1.03	2.36 ± 1.34	0.242	2.57 ± 1.19	2.48 ± 1.17	0.387

Numbers are expressed as mean ± standard deviation. TC, total cholesterol; LDL-C, low-density lipoprotein cholesterol; HDL-C, high-density lipoprotein cholesterol; TG, triglyceride.

**Table 4:** Adverse reactions.

Adverse reaction	N (%)
Statin associated muscle symptom	4 (0.37)
Liver enzyme elevation	2 (0.18)
Gastrointestinal trouble	4 (0.37)
Headache or dizziness	4 (0.37)
Arthralgia	2 (0.18)
Bruise	1 (0.09)
Impotence	1 (0.09)
Skin rash	1 (0.09)
Hyperglycemia	1 (0.09)
Total	20 (1.86)

## Discussion

In this study, we compared the efficacy and safety among 3 groups with different LDL-C lowering therapies (moderate intensity statin, high-intensity statin and statin + ezetimibe) in Korean patients with LDL-C ≥ 190 mg/dL for primary prevention. Main findings of this study are as follows: 1) although the LDL-C reduction effect was greatest in the high-intensity statins (56.0% reduction of LDL-C at 6 months), moderate-intensity statins also showed strong LDL-C lowering effects comparable to those of high-intensity statins (48.0% reduction of LDL-C at 6 months), 2) the incidence of side effects related to statin use, such as statin associated muscle symptom and liver enzyme elevation, was higher in the high-intensity group and 3) clinical outcomes were similar among 3 groups.

### Comparisons with previous studies

It has been well established that patients with LDL-C ≥ 190 mg/dL have a significant increase in the incidence of cardiovascular events and mortality [15–18], and that LDL-C lowering therapy with statin can effectively reduce their cardiovascular risk [15–18]. Based on this evidence, recent guideline recommends the use of high-intensity statin for an LDL-C reduction of ≥ 50% from baseline in these high-risk population [5]. However, all of the existing studies referenced in the guidelines are all Western-centered data [15–18]. Therefore, it may be unreasonable to apply these guidelines directly to Asians whose genetic background, body size and living environment are different from those of Europeans or North Americans. Indeed, there have been reports indicating the efficacy and safety of statin in Asians differ from those of Europeans or North Americans. In those studies, it has generally been suggested that Asians can obtain sufficient effects with lower strength statins than Europeans or North Americans [6–13]. In a study involving 6 centers in Asia, 10 mg of atorvastatin used for 8 weeks lowered LDL-C by 43% compared to baseline [13]. In a large study of 51,321 patients with hypercholesterolemia conducted in Japan, 5 mg of simvastatin lowered LDL-C by 29% during the 6-year follow-up period, which is as effective as a dose of 20 mg used in Western countries [12]. In the Management of Elevated Cholesterol in the Primary Prevention of Adult Japanese (MEGA) study, the first trial to evaluate the clinical outcomes of statin therapy in Asians, showed that using low-intensity pravastatin 10–20 mg reduced cardiovascular risk by 33% [11], which was similar to results of Western studies using higher statin doses [19, 20]. With this background, the question will naturally arise as to whether high-intensity statins should be used for Asians with LDL-C ≥ 190 mg/dL. Patients with LDL-C ≥ 190 mg/dL are at increased risk of atherosclerotic cardiovascular events [5]. The ACC/AHA guidelines defined patients with LDL-C ≥ 190 mg/dL as one of four groups requiring high-intensity statin treatment [5]. Because it has great clinical significance, it is essential to address the Asian issue for those with LDL-C ≥ 190 mg/dL. However, to the best of our knowledge, only 1 Asian study has addressed this issue. Kim *et al*. conducted a study by retrospectively reviewing the medical records of 179 Korean patients with LDL ≥ 190 mg/dL, and showed that LDL-C reduction rates did not differ between the moderate- and high-intensity statin groups [7]. In contrast to this study, our results showed that high-intensity statins were more potent in reducing LDL-C compared to moderate-intensity statins. This may be because the statistical significance could be obtained by enrolling a much larger number of patients. Our study also has strength because it analyzed the incidence of clinical events.

On the other hand, there are some results of Asian studies indicating that greater benefits can be obtained by increasing statin dosage [21, 22]. Lipid profiles significantly improved when 10 mg of rosuvastatin was increased to 20 mg in patients with heterozygous familial hypercholesterolemia in Japan [22]. In a study of patients with documented cardiovascular disease in 5 Asian countries, the LDL-C target reached 72% when simvastatin 20 mg was used, whereas the LDL-C target reached 94% when a of 80 mg dose was used [21]. These studies imply that high-intensity statin use may be necessary in some patients with severe hypercholesterolemia. Through a well-designed randomized control study, it is necessary to draw more clear conclusions by comparing the efficacy and side effects between high-intensity and moderate-intensity statins in patients with LDL ≥ 190 mg/dL.

### Clinical implications

Since beneficial effects of statin is greater than side effects, high-intensity statins are actively recommended for high-risk patients [5, 23]. However, in the case of similar efficacy, a lower-intensity statin should be selected to reduce the incidence of side effects and increase patient adherence. Asian studies have reported that the efficacy of moderate-intensity statins is as effective as those of Europeans or North Americans even in high-risk patients [6–13]. Our study of Koreans also showed that although moderate-intensity statin therapy had a less potent LDL-C lowering effect than high-intensity statin therapy, the difference in LDL-C lowering degree was not clinically significant, 56% versus 48%. Even with moderate-intensity statin therapy, it is possible to lower LDL-C by nearly 50% compared to the baseline suggested in the guidelines. Also, compared to high-intensity statins, moderate-intensity statins were as effective in preventing cardiovascular events and have lower side effects in high-risk patients with LDL-C ≥ 190 mg/dL. Even if there is severe hypercholesterolemia (LDL-C ≥ 190 mg/dL), Asians do not necessarily need to select a high-intensity statin from the beginning. Instead, it can be recommended to initially use a moderate-intensity statin, and then select appropriate statin intensity according to the degree of LDL-C reduction and side effects in this high-risk population. However, there is one caveat in interpreting our findings, particularly concerning the occurrence of clinical events. In our study, the incidence of composite events, including death, myocardial infarction, and stroke, was very low at 3.8% during the median follow-up period of 2.23 years. In Korean patients with LDL > 190 mg/dL, moderate-intensity statin therapy may be effective and safe, at least for this relatively short clinical follow-up period. A prospective study with long-term follow-up is needed to secure the justification for moderate-intensity statin use in patients with LDL-C > 190 mg/dL.

Our study also showed that the statin + ezetimibe, low-intensity statin and moderate-intensity stating groups produced similar effects as the high-intensity statin group, and had fewer side effects. Although there was no statistical difference, statin + ezetimibe group showed greater LDL-C reduction than the moderate-intensity statin group. Based on these results, it is also worth considering of a combination of statin and ezetimibe instead of high-intensity statins in patients with LDL-C ≥ 190 mg/dL.

### Study limitations

The current study has several limitations. First, although efforts were made to obtain accurate information on the occurrence of adverse events or clinical events related to statin use, some events may have been missed in this retrospective study. Second, despite a relatively large number of enrolled patients, there was a possibility that the number of clinical events was too small to reach a statistical difference among the 3 groups. There is a risk of type 2 error because it is a retrospective study and power analysis was not performed. The reasons for the low incidence of cardiovascular events in our study may be that high-risk patients with a history of previous cardiovascular disease were excluded, and that LDL-C was lowered by ≥ 48% by statins in all study patients. Third, there may have been changes in statin types or doses during clinical follow-up in some patients, but this was not properly reflected in the study. Fourth, given that baseline higher LDL-C level is associated with better statin response [24], the LDL-lowering effect might be enhanced in high-intensity statin groups due to the higher baseline LDL-C levels compared to moderate-intensity statin group. Fifth, because of unavoidable limitations of retrospective study, it was not possible to standardize treatment strategies among doctors. Lastly, since the patients analyzed in this study are all Koreans, it is difficult to apply our results to other ethnic groups.

However, despite these shortcomings, our study has several strengths. Our study sample size is relatively large. We also provided Asian data in patients with LDL ≥ 190 mg/dL, which has been scarcely reported. Another advantage of our study is that it presented outcome data on cardiovascular events as well as side effects associated with statin use.

### Conclusions

Compared to high-intensity statin therapy, moderate-intensity statin therapy was less potent in reducing LDL-C, but was effective in reaching LDL-C target in Korean patients with LDL-C ≥ 190 mg/dL. Moderate-intensity statin therapy was as effective as high-intensity statin therapy in protecting cardiovascular events. Also, moderate-intensity statin therapy had fewer side effects than high-intensity statin therapy. Therefore, it can be optional to start with a moderate-intensity statin in this high-risk population. Further prospective studies with large sample sizes are needed to confirm our findings.

## Supporting information

S1 Data (XLSX)
